# Radiative transfer model inversion using high-resolution hyperspectral airborne imagery – Retrieving maize LAI to access biomass and grain yield

**DOI:** 10.1016/j.fcr.2022.108449

**Published:** 2022-06-01

**Authors:** Ahmed Kayad, Francelino A. Rodrigues, Sergio Naranjo, Marco Sozzi, Francesco Pirotti, Francesco Marinello, Urs Schulthess, Pierre Defourny, Bruno Gerard, Marie Weiss

**Affiliations:** a Department TESAF, University of Padova, Viale dell’Università, 16, 35020 Legnaro, PD, Italy; b Agricultural Engineering Research Institute (AEnRI), Agricultural Research Centre, Giza 12619, Egypt; c CIMMYT-Mexico, Texcoco 56237, Mexico; d Lincoln Agritech Ltd, Lincoln University, Lincoln CP 7674, New Zealand; e CIMMYT China Collaborative Innovation Center, Henan Agricultural University, Zhengzhou 450002, China; f Earth and Life Institute, Université Catholique de Louvain, Croix du Sud 2 L5.07.16, 1348 Louvain-la-Neuve, Belgium; g AgroBioSciences Department, Mohammed VI Polytechnic University, Ben Guerir 43150, Morocco; h INRAE EMMAH, UMR 1114, 84914 Avignon, France

**Keywords:** Precision agriculture, PROSAIL, Vegetation indices, Maize within-field variability, Digital farming

## Abstract

Mapping crop within-field yield variability provide an essential piece of information for precision agriculture applications. Leaf Area Index (LAI) is an important parameter that describes maize growth, vegetation structure, light absorption and subsequently maize biomass and grain yield (GY). The main goal for this study was to estimate maize biomass and GY through LAI retrieved from hyperspectral aerial images using a PROSAIL model inversion and compare its performance with biomass and GY estimations through simple vegetation index approaches. This study was conducted in two separate maize fields of 12 and 20 ha located in north-west Mexico. Both fields were cultivated with the same hybrid. One field was irrigated by a linear pivot and the other by a furrow irrigation system. Ground LAI data were collected at different crop growth stages followed by maize biomass and GY at the harvesting time. Through a weekly/biweekly airborne flight campaign, a total of 19 mosaics were acquired between both fields with a micro-hyperspectral Vis-NIR imaging sensor ranging from 400 to 850 nanometres (nm) at different crop growth stages. The PROSAIL model was calibrated and validated for retrieving maize LAI by simulating maize canopy spectral reflectance based on crop-specific parameters. The model was used to retrieve LAI from both fields and to subsequently estimate maize biomass and GY. Additionally, different vegetation indices were calculated from the aerial images to also estimate maize yield and compare the indices with PROSAIL based estimations. The PROSAIL validation to retrieve LAI from hyperspectral imagery showed a R^2^ value of 0.5 against ground LAI with RMSE of 0.8 m^2^/m^2^. Maize biomass and GY estimation based on NDRE showed the highest accuracies, followed by retrieved LAI, GNDVI and NDVI with R^2^ value of 0.81, 0.73, 0.73 and 0.65 for biomass, and 0.83, 0.69, 0.73 and 0.62 for GY estimation, respectively. Furthermore, the late vegetative growth stage at V16 was found to be the best stage for maize yield prediction for all studied indices.

## 1 Introduction

Maize (Zea mays) is one of the major food crops over the world, cultivated on more than 182 million ha and producing over 1400 million ton of grain in 2018 with an average grain yield of 7.7 ton/ha (FAO, 2020). The demand for food is increasing worldwide and climate change aggravates the volatility of yield (Cogato et al., 2019). Crop yield varies between seasons, countries, fields, varieties and even within the same field due to diverse management practices and environmental conditions. Monitoring this within-field variability in season and from previous seasons provides a piece of essential information for farmers, land rentals and insurance companies for decision making. Furthermore, within-field variability of yield from previous season is one of the fundamental inputs for site-specific crop management recommendations, such as fertilizer and seed rates (Kayad et al., 2021) through the delineation of management zones.

A common practice for site-specific crop management is to collect information with remote and proximal sensing to investigate within-field variability of different factors that drive crop yield and quality. Acquired images through this approach require a proper analysis with specific algorithms, in order to generate the appropriate information layers for subsequent decision making and calculation of optimal input rates. Use of unmanned aerial vehicles is increasing rapidly in agriculture. They provide images with a high spatial resolution covering up to hundreds of hectares in a single flight (Caballero et al., 2020, Lan et al., 2010). These images can also be used as a tool for proving concepts at experimental plot scale for later being upscale through satellite imagery, which can cover regional areas.

Leaf Area Index (LAI) is an important biophysical variable for monitoring maize growth and estimating yield (Su et al., 2019a). LAI is a dimensionless (m^2^/m^2^) variable that describes the canopy structure and is related to the vegetation photosynthetic activity and plant health. It can be considered as a potential proxy for crop biomass, harvest index and grain yield (Baret et al., 1989, Haboudane et al., 2004, Jonckheere et al., 2004, Weiss et al., 2004). Crop LAI measurements are based on different techniques that can be split between direct and indirect methods (Strachan et al., 2015). Direct methods require plant leaves collection to be measured by leaf scanner instrument. Indirect methods, e.g. close range detection techniques, such as ceptometers, digital hemispherical photography (fish-eye), smartphone applications and remotely sensed data (Confalonieri et al., 2013, Jonckheere et al., 2004, Weiss et al., 2004) which measure the reflectance from incident-diffuse and/or the direct illumination, are also widely recognized for such measurements (Facchi et al., 2010, Francone et al., 2014). Destructive methods are more accurate, however more labouring and time consuming as compared to indirect methods. Furthermore, direct methods and close range techniques are both limited in terms of spatial sampling, while remotely sensed techniques allow for an exhaustive characterization of the fields of large spatial extent.

Estimating LAI with remote sensing (RS) data provides a non-destructive, rapid and cost-effective method over large areas, allowing also frequent measurements. Two main approaches for LAI estimation from RS data are commonly used. First, the empirical approach is based on fitting relationships between remotely sensed canopy reflectance through spectral vegetation indices (VIs) and in situ LAI measurements (Haboudane et al., 2004, Qiao et al., 2020, Towers et al., 2019). The second approach exploits the knowledge of the physics of the interaction between electromagnetic radiation and vegetation surfaces, developed using radiative transfer theory (Campos-Taberner et al., 2016, Danner et al., 2019, Delloye et al., 2018, Duan et al., 2014, Houborg et al., 2009, Jacquemoud et al., 2000, Mananze et al., 2018, Punalekar et al., 2018). Radiative transfer models (RTM) allow for the simulation of reflectance from a set of biophysical variables. Inverting these models from reflectance measurements then allow for estimating these biophysical variables (Darvishzadeh et al., 2008, Mananze et al., 2018). The empirical approach through VIs is simple and fast, while it cannot be easily generalized as it depends on the season, location, crop density, plant species, growth stage and specific spectral sensor resolution (Rivera et al., 2014, Su et al., 2019a). Furthermore, it does not allow for exploiting all the relevant spectral information compared to RTM, as only two or three bands are mostly used for VIs calculation (Baret and Buis, 2008, Berger et al., 2018). On the other hand, the RTM inversion is more robust as the physical laws governing the radiative transfer model within the canopy are not site or date specific but depend on the observation and illumination geometry, besides the biochemical and structural properties of the vegetation elements that are described by the input biophysical variables to be estimated (Darvishzadeh et al., 2008, Mananze et al., 2018). However, RTM may require a fairly complex process to well represent the canopy structure related to a given crop while the accuracy of the model estimation depends on the predefined model parameters and natural uncertainties of the model, which is commonly referred to by the ill-posed inverse problem (Combal et al., 2003, Houborg et al., 2015).

VIs play an important role in monitoring variation in vegetation and are defined as the arithmetic combination of two or more spectral bands, which allows for enhancing vegetation while minimizing background effects (Matsushita et al., 2007). Leaf reflectance in the visible spectral range is mostly affected by the leaf chlorophyll content. Chlorophyll shows a higher reflectance in the green (G) spectrum region compared to red (R) and blue (B) regions (Barnes et al., 2015). At canopy level in the near-infrared (NIR) region, the reflectance is much greater than in the visible due to light scattering caused by interactions between leaf internal structure and the incident radiation (Knipling, 1970). The narrow portion between R and NIR regions is called Red-Edge (RE), where vegetation reflectance increases dramatically compared to a smaller increase in the case of soils and other terrestrial objects (Corti et al., 2018, Scotford and Miller, 2005). Vegetation indices exploit such differences in leaf and canopy reflectance properties in the visible and near-infrared spectrum according to the variable of interest.

The use of remotely sensed data and its derived VIs (Kayad et al., 2019, Schwalbert et al., 2018) or assimilation in crop growth models for monitoring crop growth (Huang et al., 2019a) and yield estimation is already well established (Bala and Islam, 2009, Bouman, 1995, Gao et al., 2018, Mkhabela et al., 2011, Monteith, 1972, Rodrigues et al., 2018, Schulthess et al., 2013). Many VIs provide a good estimate of the accumulated absorbed photosynthetically active radiation (APAR) which in turn controls biomass accumulation (Akitsu et al., 2017, Monteith, 1972). For instance, the normalised difference vegetation index (NDVI) (Hasegawa, 1976) is widely used for crop growth and yield estimations, however it is limited by some saturation for medium to high LAI values (Hatfield et al., 2008, Peralta et al., 2016, Rembold et al., 2013). Other VIs show better sensitivity for high LAI values such as the green normalized difference vegetation index (GNDVI) (Gitelson et al., 1996) or the normalized difference red-edge (NDRE) (Hatfield et al., 2008). Several studies investigated the possibility of estimating maize biomass and grain yield (GY) through VIs (Kayad et al., 2019, Madugundu et al., 2017, Schulthess et al., 2013, Schwalbert et al., 2018, Venancio et al., 2020) due to their high correlation with several biophysical variables and simplicity (Ji and Peters, 2007, Lambert et al., 2018).

Kayad et al. (2019) investigated the possibility to monitor maize GY using Sentinel-2 satellite images through different VIs and machine learning techniques. Their results showed that GNDVI was the best index to describe within-field GY variability at the R4-R6 growing stage with R^2^ values of up to 0.48 for GNDVI and up to 0.6 from random forest correlation based model. Shanahan et al. (2001) suggested GNDVI at the mid-grain filling stage for maize GY prediction in plot experiment. Furthermore, Schwalbert et al. (2018) reported in a recent study that NDRE, GNDVI and NDVI showed high performance in maize yield prediction from Sentinel-2 images collected ± 20 days from the flowering stage. In general, monitoring crop yield variability through empirical models derived from VIs is already well investigated while it is only valid under specific conditions, requires field measurements concurrent to remote observations and tends to have spatial and temporal limitations (Berger et al., 2018, Hatfield et al., 2008).

In this study, we explored the inversion of the PROSAIL RTM (Jacquemoud et al., 2009), which is a combination between the leaf optical properties model (PROSPECT) with the scattering by arbitrary inclined leaves model (4-SAIL). PROSPECT is a key model to simulate leaf reflectance over the whole optical domain and comes with different versions, such as PROSPECT 4, 5 and D (Baret et al., 1992, Jacquemoud and Baret, 1990). The 4-SAIL model was proposed by Verhoef (Verhoef, 1985, Verhoef, 1984) to simulate bidirectional reflectance of a canopy (Jacquemoud et al., 2009). Previous studies on PROSAIL investigated its ability to retrieve biophysical and biochemical variables such as LAI and chlorophyll from maize, wheat, rice, sugar beet, potato and grassland (Atzberger et al., 2015, Baret et al., 2007, Darvishzadeh et al., 2012, Darvishzadeh et al., 2008, Duan et al., 2014, Herrmann et al., 2011, Punalekar et al., 2018, Richter et al., 2011, Richter et al., 2009, Sehgal et al., 2016). In some cases, LAI and chlorophyll retrievals were followed by yield or nitrogen content estimations using empirical equations or crop-growth models (Cheng et al., 2016, Huang et al., 2019b, Jay et al., 2017, Punalekar et al., 2018, Zhang et al., 2016). Comprehensive systematic reviews were done by Jacquemoud et al. (2009) and Berger et al. (2018) reporting PROSAIL theory, applications and evaluation for future capabilities.

Although several studies focused on maize LAI, biomass and GY estimation through different remote sensing approaches, there is a need to evaluate the use of the PROSAIL approach on actual maize farmer fields dealing with natural field spatial variability for commercial applications. Furthermore, comparing PROSAIL with simple empirical VI approach is needed to understand their relative strengths and weaknesses. This study has the following specific objectives:

(1) perform PROSAIL model inversion for maize LAI retrieval at different crop growth stages;
(2) assess maize biomass and GY relationships with retrieved LAI from PROSAIL;
(3) compare the relationships between selected VIs and retrieved LAI with maize ground biomass and GY.

## 2 Materials and methods

### 2.1 Study area and ground data collection

**Fig. 1:**
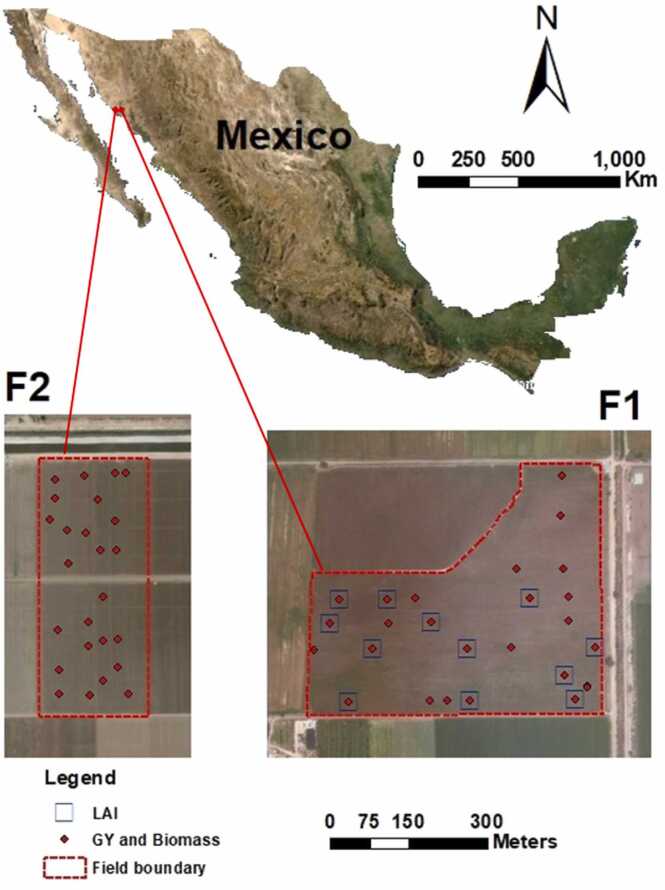
Study fields from north-west Mexico and location of ground measurements: F1 and F2.

This study was conducted in two separate fields (23 km distance) located in the Yaqui Valley near Ciudad Obregón (Sonora), in north-western Mexico (Fig. 1). Those fields were cultivated with the same maize variety (Caribú) during the same growing season 2014–2015. The first field (27°17'N, 109°57'W) named F1 which has an area of 20 ha. It was sown on October 4th, 2014 with a seed rate of 10 seeds / m^2^. The second field (27°26'N, 110°07'W) named F2 has an area of 12 ha and was sown on September 20th, 2014 with a seed rate of 9 seeds / m^2^. Row distance was 0.8 m. F1 was irrigated by a furrow irrigation system (flood), while for F2, a linear pivot irrigation system was used. Residues from the previous crop of F1 were burned (maize), while they were incorporated into the soil using a conventional tillage for F2 (potato). Climate in the study area is semi-arid with an average rainfall of 280 mm per year and soil types are clayey and alluvial soils at 3:2 ratio (Meisner et al., 1992, Rodrigues et al., 2018).

Leaf Area Index (LAI) were measured on the ground using the LAI ACCUPAR LP-80 developed by METER Group, Inc. USA. Measurements were made at five different maize growth stages between 40 and 157 days after sowing (DAS). The LAI data were collected at 12 fixed ground points which resulted in a total of 60 ground LAI observations for F1. Each observation consisted of three readings made perpendicular to the two central rows along the sampling area, which is described in the next paragraph. For the subsequent analyses, the averages of the three readings were used. The LAI measurement considered the solar zenith angle at each field location, observation date and time calculated from the National Oceanic and Atmospheric Administration (NOAA) website (https://www.esrl.noaa.gov/gmd/grad/solcalc/azel.html).

Biomass and GY were sampled at the harvesting time at 26 and 25 sampling points from F1 and F2 fields, respectively, including the 12 LAI ground points above mentioned from F1 field (Fig. 1). Biomass and GY were sampled from the 2 rows adjacent to the centre of each sampling point. Sampling distance for each row was 5 linear metres, resulting in a sampling area of 8 m^2^. The final observations were calculated in dry tons ha^-1^. Biomass refers to the whole above ground plant material harvested per unit area while grain yield refers to weighted harvested grain per unit area.

### 2.2 Hyperspectral airborne flight campaign and image processing

Aerial hyperspectral images were collected from the two fields at 12 different dates - varying from V7 to R6 growth stages (GS) (Hanway, 1963). A total of 9 images from field F1 and 10 images from field F2 were collected. From those, five images for F1 had simultaneous measurements of ground LAI. Table 1 show dates of observations with the corresponding DAS and GS from each field.

**Table 1:** Collected data from study fields.

I	Date	F1				F2		
		Image	LAI	DAS	GS	Image	DAS	GS
1	24/10/2014					✓	34	V7
2	07/11/2014					✓	48	V8
3	13/11/2014	✓	✓	40	V7		54	V9
4	26/11/2014	✓	✓	53	V9	✓	67	V16
5	10/12/2014	✓		67	V16	✓	81	V18
6	17/12/2014	✓	✓	74	V16	✓	88	V18
7	05/01/2015					✓	107	R1
8	10/01/2015	✓		98	V18	✓	112	R1
9	19/01/2015	✓	✓	107	VT	✓	121	R2
10	04/02/2015	✓	✓	123	R2	✓	137	R3
11	25/02/2015	✓		144	R4	✓	158	R6
12	10/03/2015	✓		157	R6			

* DAS: Day After Sowing.

*GS: Growth Stage.

The aerial hyperspectral imagery flight campaign was carried out with a push-broom micro-hyperspectral imaging sensor model, Micro-Hyperspec VNIR (Headwall Photonics, Bolton, MA, USA). This sensor measures the reflectance within the spectral region of 400–850 nm, split into 250 channels. The sensor was mounted on a manned airplane flying at 350 m above ground yielding images of 0.7 m ground sampling distance. We used crosses, made of 3 m long sheets of polyethylene coated with aluminium foil, as ground control points and placed them around the study sites and measured their coordinates with a real-time kinematic (RTK) GNSS model, Trimble R4 GNSS system (Trimble, CA, USA). They were subsequently used for geo-referencing of the hyperspectral images. The root mean square error of georeferencing was less than the pixel size (0.7 m). During the processing, the geolocation of each image was done using ENVI 5.6 version (ENVI, Research Systems Inc., Boulder, CO, USA).

Based on the methodology described by Rodrigues et al. (2018), the hyperspectral sensor had been radiometrically calibrated in the laboratory using an integrating sphere, CSTM-USS-2000 C Uniform Source System (LabSphere, NH, USA) at six integration times and four levels of illumination. Hyperspectral imagery was atmospherically corrected using the total incoming irradiance at 1 nm intervals simulated with the SMARTS model hosted by the National Renewable Energy Laboratory, US Department of Energy (Gueymard, 2005, Gueymard, 1995). The aerosol optical depth was measured at 550 nm with a Micro-Tops II sun photometer model (Solar LIGHT Co., Philadelphia, PA, USA) at the time of the flights. The SMARTS model computes clear sky spectral irradiance, including hemispherical diffuse, direct beam, circumsolar and total irradiance on a tilted or horizontal plane for specified atmospheric conditions. The algorithms were developed to match the output from the MODTRAN complex band models to within 2%, using aerosol optical depth as an input. The spectral resolution was 1 nm for the 400–1750 nm and 0.5 nm for the 280–400 nm ranges of the electromagnetic spectrum. This radiative transfer model had been previously used for the atmospheric correction of narrow-band multispectral imagery in several studies (Berni et al., 2009, Calderón et al., 2015, Calderón et al., 2013, Zarco-Tejada et al., 2016, Zarco-Tejada et al., 2012).

**Fig. 2:**
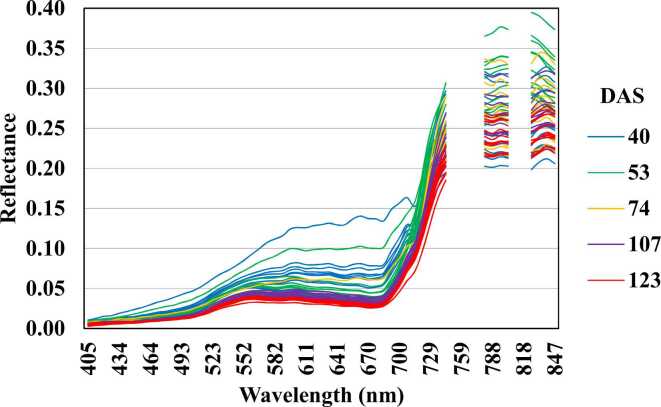
Spectral data corresponding to the 60 ground LAI measurements at different DAS from field F1.

Spectral binning was performed on each mosaic into 7.5 nm FWHM (Full Width at Half Maximum) to decrease noise effects, resulting in 61 wavelengths. From those, the 751, 759, 766, 773, 810 and 818 nm wavelengths were removed due to oxygen absorption by the sensor and noise effects. Finally, 55 wavelengths were used for subsequent analyses. All mosaics were used for data extraction based on the ground measurements locations (26 points from F1 and 25 from F2) with a buffer of 3 m diameter from the centre of the geocoordinates. This buffer distance allowed for averaging of about 7 pixels from each point. Finally, all spectral data were associated with the corresponding maize biomass, GY and matched ground LAI observations for further analyses. Fig. 2 shows the spectral data acquired across five hyperspectral images, which corresponds to the 60 ground LAI measurements.

### 2.3 PROSAIL inversion for LAI retrieval

To build the PROSAIL model, a combination between the PROSPECT-4 and 4-SAIL models was used. In the PROSPECT-4 model, four leaf parameters are required to build the model: leaf structure (N), chlorophyll a+b content (C_ab_), water thickness (C_w_) and dry matter content (C_m_). Whereas the 4-SAIL model requires the following parameters: leaf reflectance and transmittance (PROSPECT output), LAI, average leaf inclination angle (ALIA) of an ellipsoidal leaf angle distribution function (Campbell, 1990, Duan et al., 2014), hot spot parameter (Hot), soil brightness (αsoil), sun zenith angle (θs), observer zenith angle (θv) and relative azimuth angle (φSV) (Berger et al., 2018).

Maize biophysical parameters to feed PROSAIL model were obtained from the literature (Berger et al., 2018, España et al., 1999, Richter et al., 2009, Verrelst et al., 2016). Rather wide, but also narrow ranges were found for some of the parameters. For instance, N ranged between 1.2 and 2, C_ab_ ranged between 20 and 80 µg/cm^2^, LAI ranged between 0.5 and 7 m^2^/ m^2^ and ALIA ranged between 30˚ to 90˚. Whilst, other crop related parameters such as C_w_ and C_m_ were in a narrower range. The remaining PROSAIL model parameters are not related to the crop biophysical variables such as: αsoil, θs, θv and φSV. Soil brightness (αsoil) values ranged between 0 and 1. This parameter depends on soil type and moisture content. Sun zenith angle (θs) depends on the location of the study area and the day of the year the study is presented. θs values were calculated from the NOAA as described in Section 2.1 for the whole growing season, with values ranging between 42˚ to 52˚. Additionally, information about viewing geometries such as θv and φSV depends on the sensor and flight information which in our case have the ranges between − 24˚ to 24˚ and 0˚ to 180˚, respectively. Global sensitivity analysis (GSA) and preliminary analysis were done to examine different combinations of PROSAIL model parameters and the influence of each parameter simulated in the final look up table (LUT). Based on this preliminary analysis and GSA, we decided to change only the most sensitive parameters which were the ones having the wide range of magnitude and sensitivity, while other parameters were kept constant. This decision seems to be reasonable considering the required computational power and the fact that this study focused on one crop within the same region.

The ARTMO software package (Rivera et al., 2014) was used to simulate a maize spectral reflectance training dataset and to perform the GSA at different leaf and canopy parameters through the PROSAIL model. Building the PROSAIL based model consisted of three steps: First, setting up the sensor characteristics, which include the number of wavelengths (55 bands), its spectral range (400–850 nm) and FWHM (7.5 nm). Second, adding maize biophysical parameters, field and flight conditions to the PROSPECT-4 and 4-SAIL models as shown in Table 2 and described previously. Finally, running the PROSAIL simulations for specified maize parameters to simulate its spectral reflectance. The parameter θs was fixed at 49˚ which is the median value at the study area through the growing season. Additionally, C_m_ and C_W_ were fixed at 0.005 g/cm^2^ and 0.02 cm, respectively, which are in line with literature range values (Berger et al., 2018). The GSA analysis results showed that no influence of these parameters in visible and NIR regions, while θv and φSV were sampled within the predefined range to cover the different observation scenarios. The PROSAIL spectral dataset was generated for five free variables N, C_ab_, LAI, ALIA and hot spot sampled within the predefined ranges using a uniform distribution function except for LAI where a latin hypercube distribution function was used. As a result, a total of 60750 simulations were obtained for the dataset.

**Table 2:** Maize parameters values for PROSAIL model.

Parameter	Symbol	Units	Range
**Leaf model: PROSPECT-4**			
Mesophyll structure index	N	Unit less	1.2–2
Chlorophyll a + b	C_ab_	µg/cm^2^	30–80
Dry matter content	C_m_	g/cm^2^	0.005
Equivalent water thickness	C_W_	Cm	0.02
**Canopy model: 4-SAIL**			
Leaf area index	LAI	m^2^/ m^2^	0.5–7
Average leaf inclination angle	ALIA	◦	35–90
Hot spot parameter	Hot	m/m	0 – 0.28
Soil brightness	Αsoil	Unit less	0–1
Sun zenith angle	θs	◦	49
Observer zenith angle	θv	◦	-24–24
Relative azimuth angle	φSV	◦	0–180

**Fig. 3:**
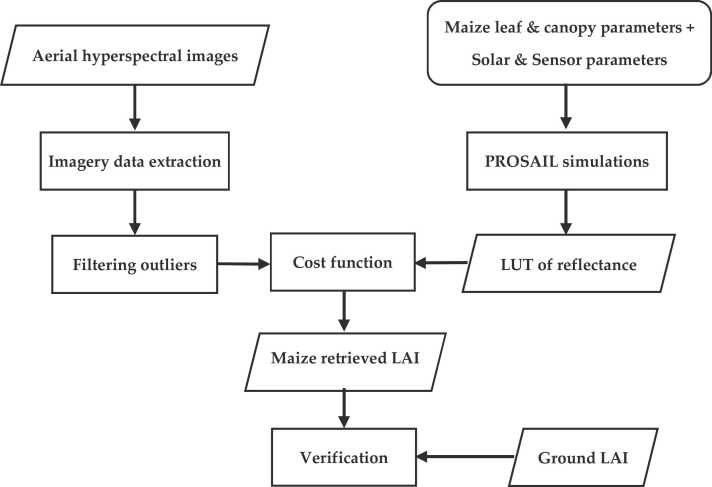
The development flowchart for Maize LAI retrieval model based PROSAIL.

Finally, the cost function was used to find the best match between the simulated data set and the available ground LAI data with the corresponding spectral data (Weiss et al., 2004). The least square error (LSE) cost function was used to select the solution of the inverse problem and the best 10% of the solutions corresponding to the smallest LSE values were averaged to calculate the modelled estimates of free parameters. Such approach were reported by several studies to reduce the influence of the ill-posed problem caused by measurement errors and model inadequacies (Duan et al., 2014). The 60 ground LAI data (12 ground sampling points from F1 time five images) were used to validate the retrieved LAI from PROSAIL. Fig. 3 illustrates the maize LAI retrieval flowchart based on PROSAIL and LUT inversion approach. The validated model was used to retrieve LAI from all remaining available ground sampling points where GY and biomass were measured, across all hyperspectral time series from both field locations. LAI data retrieved from PROSAIL model inversion will be referred as ‘retrieved LAI’ hereafter.

### 2.4 Calculated VIs

In order to compare the estimation of biomass and GY by the retrieved LAI from PROSAIL inversion with a simpler approach as VIs, three VIs were calculated; NDVI, GNDVI and NDRE ((1), (2), (3)). These VIs were chosen based on their simplistic forms and previous results shown in the literature (Kayad et al., 2019, Peralta et al., 2016, Schwalbert et al., 2018). VIs were calculated from all acquired aerial hyperspectral images from both study fields and extracted using the 51 ground point locations from both study fields (Fig. 1).

(1) $$\mathrm{NDVI}=\frac{\mathrm{NIR}-R}{\mathrm{NIR}+R}$$

(2) $$\mathrm{GNDVI}=\frac{\mathrm{NIR}-G}{\mathrm{NIR}+G}$$

(3) $$\mathrm{NDRE}=\frac{\mathrm{NIR}-\mathrm{RE}}{\mathrm{NIR}+\mathrm{RE}}$$

Where: NDVI, is the normalized difference vegetation index, GNDVI is the green normalized difference vegetation index, NDRE is the normalized difference red-edge, NIR is the reflectance at the near infrared band (832 nm), R is the reflectance at the red band (663 nm), G is the reflectance at the green band (560 nm) and RE is the reflectance at the red-edge band (722 nm).

### 2.5 Data analysis

To build empirical models between the retrieved-LAI and studied VIs versus maize biomass and GY, different regression models were tested, including linear, exponential, power and logarithmic. As per this step results and the distribution of data points, regression models were fitted between maize biomass and GY versus retrieved LAI and vegetation indices. This step considered only the 12 sampling points corresponding to the ground LAI measurements from F1 (Fig. 1). A time series analysis through coefficient of determination (R^2^) values was used to assess the best crop growth stage for maize biomass and GY estimation. The subsequent analyses were done using only the best crop growth stage selected.

The empirical equations fitted in the previous analysis were retrieved by considering only the ground 12 observation points from F1, and applied to the remaining dataset. The remaining dataset, totalling 39 observations across both fields (14 from F1 and 25 from F2), was used for the cross-validation of the empirical equations. Furthermore, comparisons of the performance achieved by the different proxies – retrieved LAI and VIs – were done by assessing statistical metrics of each cross-validation. R^2^ values, root mean square error (RMSE) and mean absolute error (MAE) where calculated for this purpose.

## 3 Results

### 3.1 Field ground data

**Fig. 4:**
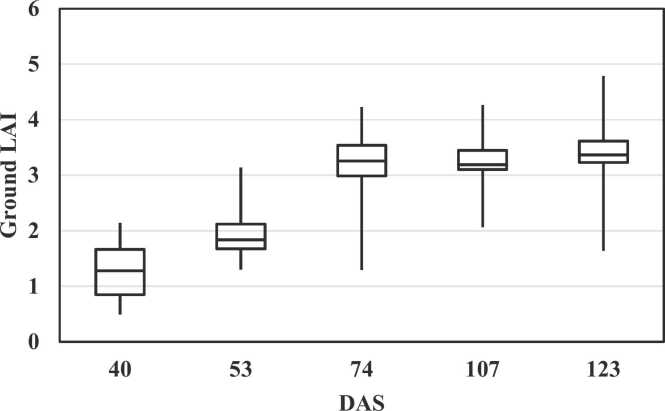
Box-plot of ground LAI observations at different crop ages for F1.

Ground LAI data were collected at different crop stages starting from 40 to 123 DAS. Boxplots for ground LAI data from F1 are shown in Fig. 4. Ground LAI data showed an average of 1.3 at 40 DAS and subsequently increased to above 3 at 74 DAS. The average LAI values across crop stages from F1 was 2.7 and reached a maximum of 4.9 at 123 DAS.

**Fig. 5:**
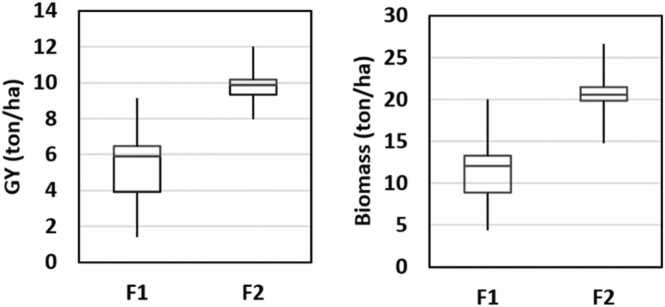
Boxplot for maize GY and biomass.

Fig. 5 shows the boxplots from maize biomass and GY. F2 produced an average of 20.5 and 9.8 ton/ha while F1 produced 11.7 and 5.4 ton/ha for maize biomass and GY, respectively. F2 yield was higher than F1 with a narrow range of variability to both, maize biomass (14.8–26.7 ton/ha) and GY (8–12 ton/ha). Whilst F1 showed a higher range variability on both, maize biomass (4.4–20 ton/ha) and GY (1.4–9.2 ton/ha). The narrow range of variability observed for F2 indicates that it was more homogeneous as compared to F1. Across both fields, the range of GY was between 1.4 and 12.0 ton/ha and for biomass, it was between 4.4 and 26.7 ton/ha. This range is quite wide, as compared to other studies (Kayad et al., 2021, Kayad et al., 2019) and global reports (FAO, 2020).

### 3.2 Retrieved LAI from PROSAIL inversion-based model

**Fig. 6:**
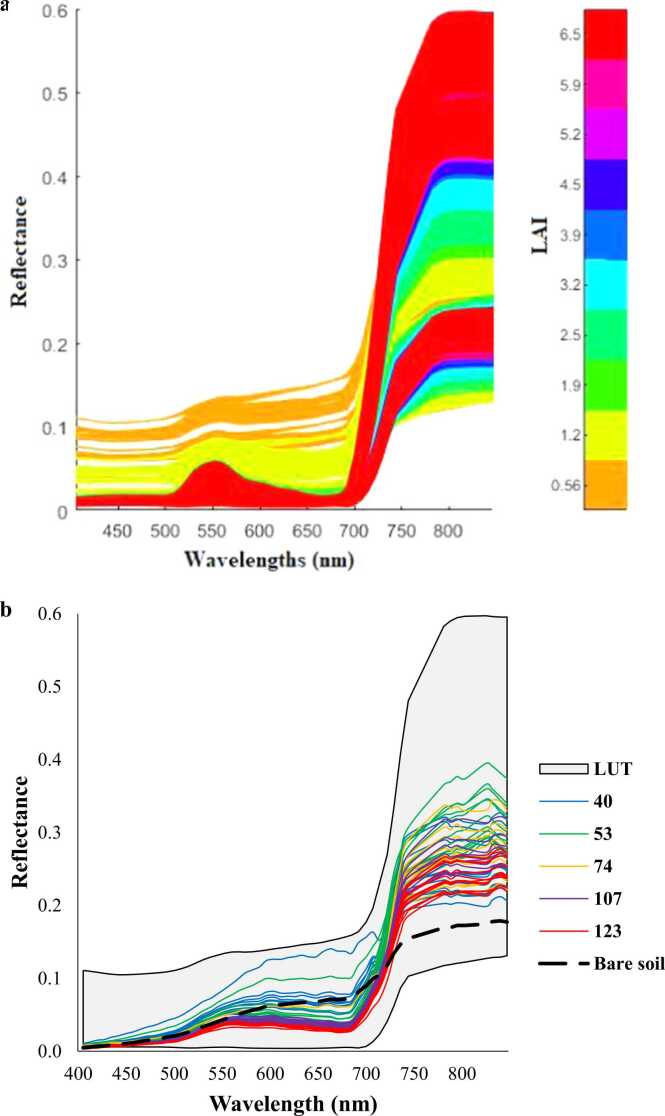
PROSAIL 60750 synthetic spectral reflectance signatures (a) and the available reflectance data at different DAS with the synthetic data range and bare soil reflectance curve from ARTMO library (b).

**Fig. 7:**
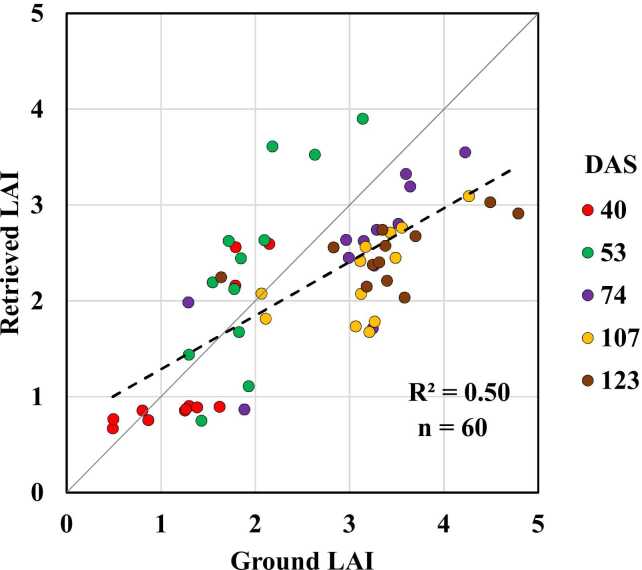
Cross-validation between ground LAI vs PROSAIL retrieved LAI at different DAS.

The PROSAIL model yielded 60750 synthetic spectral signatures using the model input parameters as per Table 2. These simulations were used through the LUT inversion approach with the LSE cost function to retrieve maize LAI from both study fields. Fig. 6, shows the 60750 synthetic spectral signatures, the measured reflectance data overlapped with the range of synthetic data and the bare soil reflectance from ARTMO library. The 60 ground LAI data points across different growth stages were used to validate the retrieved LAI from PROSAIL. Validation results showed an R^2^ value of 0.5 with a mean absolute error (MAE) of 0.7 and RMSE of 0.8 (Fig. 7). Specific maize growth stages R^2^ values were 0.63, 0.64, 0.59, 0.4 and 0.37 for 40, 53, 74, 107 and 123 DAS, respectively. This model was used to retrieve LAI from all acquired hyperspectral images where ground sampling measurements from both study fields took place, which were considered for further analysis.

### 3.3 VIs and retrieved LAI vs maize yield and biomass

**Fig. 8:**
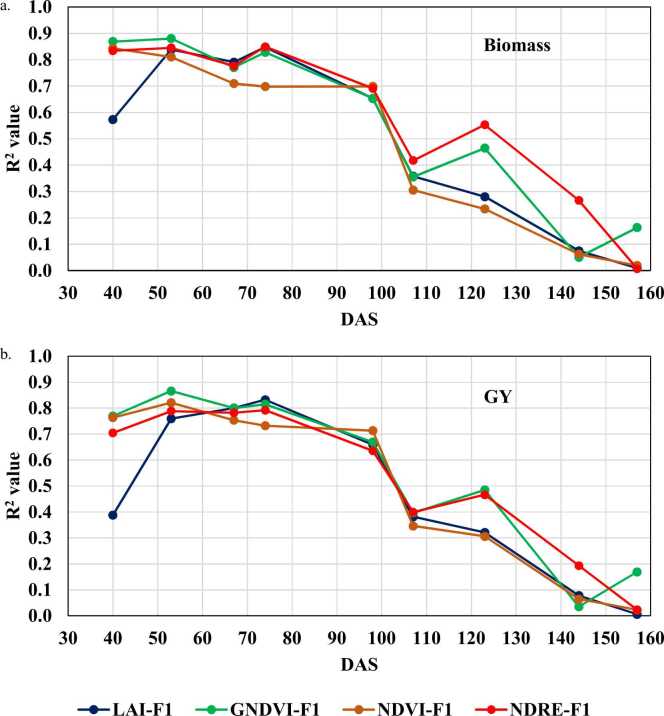
Time series of R^2^ values between VIs, LAI against maize biomass (a) and GY (b).

Calculated vegetation indices and retrieved LAI from the 12 selected ground sampling points were correlated with maize biomass and GY to describe their relationship through maize growth stages from F1. The time-series of R^2^ values between NDVI, GNDVI, NDRE and retrieved LAI versus maize biomass and GY are shown in Fig. 8a and Fig. 8b, respectively. In general, all VIs and retrieved LAI have the same trend over different growth stages. R-square started with high values (>0.65) between 40 and 98 DAS for both, biomass and GY, then declined gradually at advanced crop growth stages. At 40 DAS, retrieved LAI showed the lowest correlation values compared to the other VIs for both biomass and GY, while all indices showed very low correlations starting from 144 DAS. At 123 DAS, GNDVI and NDRE showed a higher accuracy than retrieved LAI and NDVI.

As both, VIs and LAI, showed the same temporal trend with maize biomass and GY according to the R^2^ time series, it can be concluded that the same late vegetative growing stage V16 (74 DAS at F1 and 67 DAS at F2) is most suitable for maize biomass and yield assessment. Equations 4–11 describe the empirical relationship between maize biomass and GY versus retrieved LAI, NDVI, GNDVI and NDRE at the V16 growth stage, as described in Section 2.5. These equations were applied on the remaining 39 observations points as part of the cross-validation process, assessing maize biomass and GY from both fields and evaluating model accuracies.

**Table:** 

Retrieved LAI	GY= 2.43 ×LAI-0.47	R^2^ = 0.83	(4)
	Biomass= 4.57 ×LAI+ 0.35	R^2^ = 0.85	(5)
NDVI	GY= 0.035e^6.45NDVI^	R^2^ = 0.92	(6)
	Biomass= 0.2725e^4.79NDVI^	R^2^ = 0.88	(7)
GNDVI	GY= 42.81 ×GNDVI-25.78	R^2^ = 0.82	(8)
	Biomass= 80.33 ×GNDVI-47.13	R^2^ = 0.83	(9)
NDRE	GY= 40.71 ×NDRE-8.11	R^2^ = 0.79	(10)
	Biomass= 78.47 ×NDRE-14.67	R^2^ = 0.85	(11)

Where biomass and GY in ton/ha and all *p-values* were ˂0.001.

**Fig. 9:**
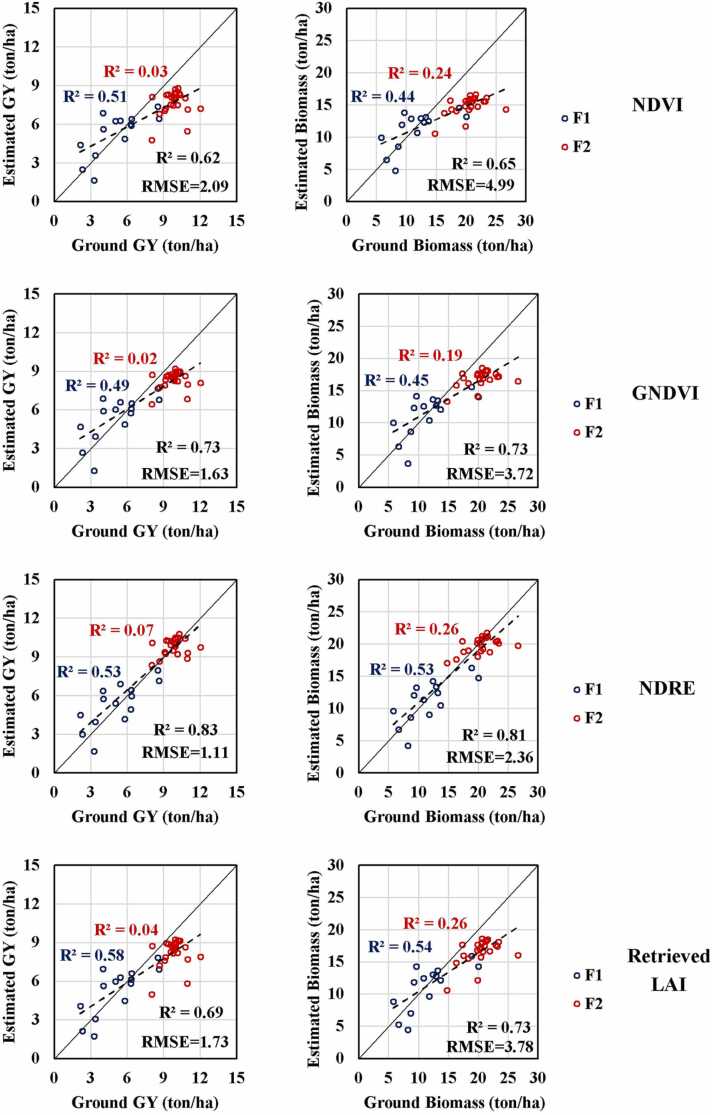
XY cross-validation graphs of estimated vs ground maize biomass and GY based on retrieved LAI and VIs empirical models.

Fig. 9 shows the XY graphs resulting from the cross-validation process, where maize biomass and GY were estimated from the VIs and retrieved LAI empirical models at V16 growth stage generated in the previous step. Estimated maize GY and biomass through NDRE showed the highest R^2^ values (0.83 and 0.81, respectively) in comparison with all tested indices, with RMSE of 1.11 and 2.36 ton/ha for maize GY and biomass, respectively. Retrieved LAI and GNDVI showed the same biomass estimation accuracy of 0.73 R^2^ value and 3.78 and 3.72 ton/ha RMSE, respectively. While GNDVI showed a slightly better performance for GY estimation. Moreover, NDVI produced the lowest estimation accuracies with R^2^ values of 0.62 and 0.65 and RMSE of 2.09 and 4.99 ton/ha for GY and biomass, respectively. Considering R^2^ values from each field, showed a quite low values from F2 due to the narrow range of variability. In F1, retrieved LAI showed the highest R^2^ values followed by NDRE and mostly equal values from GNDVI and NDVI.

## 4 Discussion

Ground LAI increased until V16 and then remained stable (Fig. 4). Feng et al. (2013) reported similar maize LAI behaviour across growth stages under drought experiments. Meanwhile, ground yield measurements from both fields showed a clear difference between them regarding spatial variability and average measurements. F2 produced 75% and 83% higher biomass and GY than F1, respectively. Furthermore, the range in variability for GY in F1 was between 1.4 and 9.2 ton/ha and 4.4–20 ton/ha for biomass. In contrary, F2 was more homogenous. Its production levels were also higher, as they ranged from 15 to 26 and 8–12 ton/ha for biomass and GY, respectively (Fig. 5). Kayad et al. (2019) reported an average maize GY of > 13.5 ton/ha in a highly productive field for continues three seasons while the FAO (2020) reported a global average of 7.7 ton/ha. These yield differences are most likely due to the different irrigation systems: F2 had more frequent post-planting irrigations and they were more evenly distributed thanks to the pivot irrigation system, whereas F1 was irrigated by a furrow irrigation system.

In this study, a total of 60750 LUT simulations based on PROSAIL input parameters were used to estimate maize LAI. This number was based on combination of different input values of N, Cab, LAI, ALIA, Hot, αsoil, θv, φSV and constant values for other model parameters according to previous studies, field and measurement conditions (Table 2). The use of identical parameters for both fields is reasonable, considering that the two fields were planted with the same hybrid and experienced the same weather conditions. Image acquisition also took place under almost identical conditions. The number of LUT simulations is in agreement with many previous studies in this field of research (Darvishzadeh et al., 2012, Duan et al., 2014, Richter et al., 2009). Duan et al. (2014) investigated different LUT sizes between 50, 100 and 250 thousand simulations to estimate LAI for sunflower, potato and maize fields and reported no significant difference between the retrieved LAI accuracies using different LUT sizes. Similar results were reported by Darvishzadeh et al. (2012) for rice chlorophyll estimations and by Richter et al. (2009) for sugar beet and maize LAI estimations. Furthermore, preliminary tests were carried out to investigate different LUT sizes by decreasing the number of steps within each parameter, reaching up to 120 thousand simulations. It was found that 60750 simulations provided reasonable results.

Retrieved LAI from PROSAIL inversion showed reasonable accuracy against ground LAI measurements where R^2^ value was 0.5 and RMSE was 0.8. This result is in agreement with previous studies from different experiments and platforms. Kimm et al. (2020) used different fused satellite datasets acquired by MODIS, Landsat and CubeSat to estimate maize LAI in the US corn belt through a PROSAIL RTM inversion. Their results showed R^2^ values of 0.69 and 0.76 while RMSE was > 1 m^2^/m^2^ between ground measured LAI and estimated LAI from STAIR fusion (MODIS-Landsat fusion) and CubeSat data, respectively. Su et al. (2019b) retrieved maize canopy LAI and chlorophyll at county scale by fusing Sentinel-2 and MODIS images through PROSAIL at four different maize growth stages. Their results showed R^2^ values of about 0.6 between ground measured and estimated LAI and chlorophyll. Su et al. (2019a) retrieved maize LAI using PROSAIL with leaf angle distribution functions generated from terrestrial laser scanning points resulting in a strong and significant correlation (R^2^ value = 0.82) between ground and estimated maize LAI.

Additionally, the retrieved versus ground LAI were relatively well distributed around the 1:1 line (Fig. 7). However, the retrieved LAI was slightly overestimated at early crop growth stages and slightly underestimated at late crop growth stages. Such a trend of over and underestimation of retrieved LAI through PROSAIL inversion was observed from previous studies (Adeluyi et al., 2021, Bacour et al., 2006, Si et al., 2012). The PROSAIL model does not take into account the shading effect of row crops (Berger et al., 2018, Dorigo, 2012, Richter et al., 2011). Duan et al. (2014) used hyperspectral images acquired by UAV in different flight directions to estimate LAI through a PROSAIL inversion. Their results showed that shading enhanced reflectance in the backward scattering direction and reduced in the forward scattering direction. Similar results were reported from multi-angular compact high resolution imaging spectrometer (CHRIS) data to estimate LAI (Verrelst et al., 2012). Another assumption could be the fact that maize is a typical row crop and affected by leaf clumping in row direction while the PROSAIL model assumes that leaves are randomly distributed within the canopy volume (Duan et al., 2014, Jacquemoud et al., 2009). Yao et al. (2008) proposed a row structure model for early maize growth stage and homogeneous canopy model for later stages to reduce the effect of row structure on LAI estimation. Moreover, in this study the spectral reflectance was extracted from each sampling point with a buffer of 3 m diameter, which is almost 7 pixels. The sampling area covers almost 4 rows which could be reasonable for reducing the row effects, especially at early stages.

According to the time series analysis for R^2^ values between Biomass and GY against retrieved LAI and VIs, the maize vegetative growth stage at V16 resulted in the highest accuracies for yield estimations. This result is in accordance with previous studies for late vegetative stages (Peralta et al., 2016, Schwalbert et al., 2018) while other studies suggested to use the grain filling stages R2-R4 for maize yield prediction (Aguate et al., 2017, Kayad et al., 2019). The difference between the studies reporting on the most suitable time for maize yield prediction could be due to the difference in experimental conditions such as maize hybrids, sensors, methodologies, scale and environmental conditions. However, most of the literature suggests the range between late vegetative to early reproductive growth stages for maize yield prediction through remotely sensed data, which is in agreement with results found in this study.

Maize biomass and GY estimation through VIs and retrieved LAI showed different accuracy levels at different growth stages. Fig. 8 shows R^2^ values up to 0.88 for biomass and 0.87 for GY at early stages till 97 DAS, then they declined gradually to < 0.2 before harvest. In general, all indices followed the same trend of high correlations at early stages, decreasing at later growth stages, while NDRE and GNDVI showed better performance than other indices at 123 DAS. This result is in agreement with Kayad et al. (2019), who suggested GNDVI for maize yield estimation at R4-R6 growth stage between 105 and 135 DAS. However, their study didn’t consider early growth stages. The lower correlation from NDVI at this specific stage could be explained by the fact of NDVI saturation at medium to high LAI values (Hanna et al., 1999, Kayad et al., 2016, Nguy-Robertson et al., 2012, Trotter et al., 2008). Additionally, for the retrieved LAI, the low range of variability at this stage could explain the low correlation at 123 DAS as shown in Fig. 4.

The selected growth stage of V16 (late vegetative stage) for yield estimation provides a balanced decision considering the findings of previous studies. Although, the R^2^ values were almost the same for the selected growth stage at 73 DAS for all studied indices except NDVI (Fig. 8), yield estimation accuracies changed as shown in Fig. 9. It is worth mentioning that in Fig. 9, NDRE estimations trend line were mostly overlaid with the 1:1 line, compared to higher deviation in case of other studied indices, which prove the robustness of NDRE approach compared to other approaches. Kayad et al. (2019) reported same indices for within-field maize yield prediction with Sentinel-2, while their R^2^ value was ˂0.5 and reached 0.6 when using machine learning techniques. Schwalbert et al. (2018) reported that NDRE retrieved from Sentinel-2 images was the most sensitive VI for maize yield prediction, followed by GNDVI and NDVI with R^2^ values ranging between 0.32 and 0.68. Additionally, NDRE outperformed GNDVI and NDVI for maize yield prediction at mid-season from high resolution RapidEye satellite imagery with R^2^ value ranging between 0.29 and 0.70 (Peralta et al., 2016). NDRE could avoid the saturation issues at medium to high LAI (Schwalbert et al., 2018). Furthermore, the higher spatial and spectral resolution of the aerial hyperspectral images used in this study could explain the better estimation accuracies than those achieved with Sentinel-2 and RapidEye satellite images.

In general, NDRE, retrieved LAI and GNDVI showed high accuracy for maize yield estimation, while NDRE outperformed the retrieved LAI for two reasons. Firstly, LAI is a biophysical variable that determines the photosynthetic active radiation (PAR), better describing the vegetative canopy structure and its photosynthetic activity. However, other biophysical variables such as chlorophyll content are also important for describing the whole canopy vigour and subsequently crop yield, whereas NDRE could provide a better proxy for the combination of both. Secondly, PROSAIL is a physically-based approach that has many approximations to simulate the biological canopy properties. Such approximations may face many challenges especially to assess within-field variability of the same crop/variety and considering the previously explained limitations of row and shading effects.

It is worth mentioning that the spatial and temporal constraints to re-apply VIs based on empirical models were reported by many previous studies (Báez-González et al., 2002, Lobell, 2013, Schwalbert et al., 2018). Although VIs showed high correlations in this study, these correlations may decrease when such empirical equations are applied to a data set that represents a wide range of conditions (Peralta et al., 2016, Schwalbert et al., 2018). On the contrary, retrieved LAI based on RTM inversion, can be applied in different fields for LAI estimation, subsequently allowing for more robust yield estimations and potentially reducing the number of in-situ measurements to a regional level assessment. Having said so, retrieving LAI from RTM inversion applied into RS imagery show its potential for accessing good crop biomass and yield proxies. However, absolute yield values will always require in-situ measurements specially from different crop varieties. The advantage of using RTM inversion is to derive an accurate and robust estimation for biophysical variables such as LAI and once calibrated, being potentially applied to different fields of the same calibrated crop for subsequently crop yield estimation.

Finally, research applying physically based radiative transfer models and testing their transferability at different spatial scales, from plot experiments to farmers’ fields, are needed to demonstrate the robustness of this approach for plant biophysical attributes retrieval. At plot experiment scales, low altitude remote sensing platforms used for high throughput phenotyping can make use of RTM models for plant traits retrieval, such as canopy chlorophyll content (Delloye et al., 2018) and carotenoids (Berger et al., 2018, Jiang et al., 2018), which can be used as proxies for resistant genotype selection under biotic and/or abiotic stresses. At farmers’ field scales, the use of such biophysical plant variables can potentially open unprecedented opportunities for within-field variability assessment of yield and quality of the product, besides supporting crop management practices related to fertilizer recommendations.

## 5 Conclusions

A field study was conducted in two different maize fields located in north-western Mexico to investigate the potential of PROSAIL model inversion to estimate LAI, for subsequently biomass and GY assessment. Maize ground LAI measured at different growth stages along with hyperspectral imagery signals were used to calibrate and validate PROSAIL and through its inversion, to retrieve LAI. Finally, retrieved LAI and VIs were used for maize biomass and GY estimations. The main findings can be summarized as follows:

1. PROSAIL model inversion was capable to retrieve maize LAI, presenting reasonable R^2^ value (0.5) and considerably low RMSE value (0.8);
2. maize yield estimation showed better performance at the late vegetative growth stages (V16), around 73 DAS;
3. maize biomass and grain yield estimations through NDRE showed higher performance in comparison with retrieved LAI, GNDVI and NDVI, when applied to an independent dataset.

## Funding

This work was funded partially by the CGIAR Research Program on Maize (www.maize.org) and by the Spurring Transformation in Agriculture Research (STARS) project under number 1094229-2014 (www.starsproject.org). We’re most grateful to Lorena Gonzalez Perez and the Crop Nutrition team for their assistance with field measurements, flight campaign and image processing; to the maize growers Rosario Caballero and Juan Escamilla, for allowing us to access to their crop fields. Much of the data analytics was conducted whilst the senior author was on a visiting scholarship with CIMMYT funded by the 10.13039/501100003500University of Padova, Italy.

## Declaration of Competing Interest

The authors declare no conflict of interest.

## References

[bib1] Adeluyi O., Harris A., Verrelst J., Foster T., Clay G.D. (2021). Estimating the phenological dynamics of irrigated rice leaf area index using the combination of PROSAIL and Gaussian Process Regression. Int. J. Appl. Earth Obs. Geoinf..

[bib2] Aguate F.M., Trachsel S., González Pérez L., Burgueño J., Crossa J., Balzarini M., Gouache D., Bogard M., de los Campos G. (2017). Use of hyperspectral image data outperforms vegetation indices in prediction of maize yield. Crop Sci..

[bib3] Akitsu T., Nasahara K.N., Hirose Y., Ijima O., Kume A. (2017). Quantum sensors for accurate and stable long-term photosynthetically active radiation observations. Agric. For. Meteorol..

[bib4] Atzberger C., Darvishzadeh R., Immitzer M., Schlerf M., Skidmore A., le Maire G. (2015). Comparative analysis of different retrieval methods for mapping grassland leaf area index using airborne imaging spectroscopy. Int. J. Appl. Earth Obs. Geoinf..

[bib5] Bacour C., Baret F., Béal D., Weiss M., Pavageau K. (2006). Neural network estimation of LAI, fAPAR, fCover and LAI×Cab, from top of canopy MERIS reflectance data: principles and validation. Remote Sens. Environ..

[bib6] Báez-González A.D., Chen P.Y., Tiscareño-López M., Srinivasan R. (2002). Using satellite and field data with crop growth modeling to monitor and estimate corn yield in Mexico. Crop Sci..

[bib7] Bala S.K., Islam A.S. (2009). Correlation between potato yield and MODIS-derived vegetation indices. Int. J. Remote Sens..

[bib8] Baret F., Buis S. (2008). Estimating canopy characteristics from remote sensing observations: Review of methods and associated problems. Adv. L. Remote Sens. Syst. Model. Invers. Appl..

[bib9] Baret F., Guyot G., Major D.J. (1989). Crop biomass evaluation using radiometric measurements. Photogrammetria.

[bib10] Baret F., Hagolle O., Geiger B., Bicheron P., Miras B., Huc M., Berthelot B., Niño F., Weiss M., Samain O., Roujean J.L., Leroy M. (2007). LAI, fAPAR and fCover CYCLOPES global products derived from VEGETATION. Part 1: principles of the algorithm. Remote Sens. Environ..

[bib11] Baret F., Jacquemoud S., Guyot G., Leprieur C. (1992). Modeled analysis of the biophysical nature of spectral shifts and comparison with information content of broad bands. Remote Sens. Environ..

[bib12] Barnes E.M., Moran M.S., Pinter P.J., Clarke T.R. (2015). Multispectral remote sensing and site-specific agriculture: examples of current technology and future possibilities. Proc. Third Int. Conf. Precis. Agric..

[bib13] Berger K., Atzberger C., Danner M., D’Urso G., Mauser W., Vuolo F., Hank T. (2018). Evaluation of the PROSAIL model capabilities for future hyperspectral model environments: a review study. Remote Sens.

[bib14] Berni J.A.J., Zarco-Tejada P.J., Suárez L., Fereres E. (2009). Thermal and narrowband multispectral remote sensing for vegetation monitoring from an unmanned aerial vehicle. IEEE Trans. Geosci. Remote Sens..

[bib15] Bouman B.A. (1995). Crop modelling and remote sensing for yield prediction. Neth. J. Agric. Sci..

[bib16] Caballero D., Calvini R., Amigo J.M. (2020). Hyperspectral imaging in crop fields: precision agriculture. Data Handl. Sci. Technol..

[bib17] Calderón R., Navas-Cortés J.A., Lucena C., Zarco-Tejada P.J. (2013). High-resolution airborne hyperspectral and thermal imagery for early detection of Verticillium wilt of olive using fluorescence, temperature and narrow-band spectral indices. Remote Sens. Environ..

[bib18] Calderón R., Navas-Cortés J.A., Zarco-Tejada P.J. (2015). Early detection and quantification of verticillium wilt in olive using hyperspectral and thermal imagery over large areas. Remote Sens..

[bib19] Campbell G.S. (1990). Derivation of an angle density function for canopies with ellipsoidal leaf angle distributions. Agric. Meteorol..

[bib20] Campos-Taberner M., García-Haro F.J., Camps-Valls G., Grau-Muedra G., Nutini F., Crema A., Boschetti M. (2016). Multitemporal and multiresolution leaf area index retrieval for operational local rice crop monitoring. Remote Sens. Environ..

[bib21] Cheng Z., Meng J., Wang Y. (2016). Improving spring maize yield estimation at field scale by assimilating time-series HJ-1 CCD data into the WOFOST model using a new method with fast algorithms. Remote Sens..

[bib22] Cogato A., Meggio F., Migliorati M.D.A., Marinello F. (2019). Extreme weather events in agriculture: a systematic review. Sustain.

[bib23] Combal B., Baret F., Weiss M., Trubuil A., Macé D., Pragnère A., Myneni R., Knyazikhin Y., Wang L. (2003). Retrieval of canopy biophysical variables from bidirectional reflectance using prior information to solve the ill-posed inverse problem. Remote Sens.Environ..

[bib24] Confalonieri R., Foi M., Casa R., Aquaro S., Tona E., Peterle M., Boldini A., De Carli G., Ferrari A., Finotto G., Guarneri T., Manzoni V., Movedi E., Nisoli A., Paleari L., Radici I., Suardi M., Veronesi D., Bregaglio S., Cappelli G., Chiodini M.E., Dominoni P., Francone C., Frasso N., Stella T., Acutis M. (2013). Development of an app for estimating leaf area index using a smartphone. Trueness and precision determination and comparison with other indirect methods. Comput. Electron. Agric..

[bib25] Corti M., Cavalli D., Cabassi G., Marino Gallina P., Bechini L. (2018). Does remote and proximal optical sensing successfully estimate maize variables? A review. Eur. J. Agron..

[bib26] Danner M., Berger K., Wocher M., Mauser W., Hank T. (2019). Fitted PROSAIL parameterization of leaf inclinations,water content and brown pigment content for winter wheat and maize canopies. Remote Sens..

[bib27] Darvishzadeh R., Matkan A.A., Dashti Ahangar A. (2012). Inversion of a radiative transfer model for estimation of rice canopy chlorophyll content using a lookup-table approach. IEEE J. Sel. Top. Appl. Earth Obs. Remote Sens.

[bib28] Darvishzadeh R., Skidmore A., Schlerf M., Atzberger C. (2008). Inversion of a radiative transfer model for estimating vegetation LAI and chlorophyll in a heterogeneous grassland. Remote Sens. Environ..

[bib29] Delloye C., Weiss M., Defourny P. (2018). Retrieval of the canopy chlorophyll content from Sentinel-2 spectral bands to estimate nitrogen uptake in intensive winter wheat cropping systems. Remote Sens. Environ..

[bib30] Dorigo W.A. (2012). Improving the robustness of cotton status characterisation by radiative transfer model inversion of multi-angular CHRIS/PROBA data. IEEE J. Sel. Top. Appl. Earth Obs. Remote Sens.

[bib31] Duan S.B., Li Z.L., Wu H., Tang B.H., Ma L., Zhao E., Li C. (2014). Inversion of the PROSAIL model to estimate leaf area index of maize, potato, and sunflower fields from unmanned aerial vehicle hyperspectral data. Int. J. Appl. Earth Obs. Geoinf..

[bib32] España M.L., Baret F., Aries F., Chelle M., Andrieu B., Prévot L. (1999). Modeling maize canopy 3D architecture: application to reflectance simulation. Ecol. Modell..

[bib33] Facchi A., Baroni G., Boschetti M., Gandolfi C. (2010). Comparing optical and direct methods for leaf area index determination in a maize crop. J. Agric. Eng..

[bib34] FAO (2020).

[bib35] Feng R., Zhang Y., Yu W., Hu W., Wu J., Ji R., Wang H., Zhao X. (2013). Analysis of the relationship between the spectral characteristics of maize canopy and leaf area index under drought stress. Acta Ecol. Sin..

[bib36] Francone C., Pagani V., Foi M., Cappelli G., Confalonieri R. (2014). Comparison of leaf area index estimates by ceptometer and PocketLAI smart app in canopies with different structures. Field. Crop. Res..

[bib37] Gao F., Anderson M., Daughtry C., Johnson D. (2018). Assessing the variability of corn and soybean yields in central Iowa using high spatiotemporal resolution multi-satellite imagery. Remote Sens..

[bib38] Gitelson A.A., Kaufman Y.J., Merzlyak M.N. (1996). Use of a green channel in remote sensing of global vegetation from EOS- MODIS. Remote Sens. Environ..

[bib39] Gueymard C.A. (2005). Interdisciplinary applications of a versatile spectral solar irradiance model: A review. Energy.

[bib40] Gueymard, C.A., 1995. SMARTS2: a simple model of the atmospheric radiative transfer of sunshine: algorithms and performance assessment. Rep. No. FSEC-PF-270–95 1–84.

[bib41] Haboudane D., Miller J.R., Pattey E., Zarco-Tejada P.J., Strachan I.B. (2004). Hyperspectral vegetation indices and novel algorithms for predicting green LAI of crop canopies: modeling and validation in the context of precision agriculture. Remote Sens. Environ..

[bib42] Hanna M.M., Steyn-Ross D.A., Steyn-Ross M. (1999). Estimating biomass for New Zealand pasture using optical remote sensing techniques. Geocarto Int..

[bib43] Hanway J.J. (1963). Growth Stages of Corn (Zea mays, L.) 1. Agron. J..

[bib44] Hasegawa S. (1976). Metabolism of limonoids. Limonin d-ring lactone hydrolase activity in pseudomonas. J. Agric. Food Chem..

[bib45] Hatfield J.L., Gitelson A.A., Schepers J.S., Walthall C.L. (2008). Application of spectral remote sensing for agronomic decisions. Agron. J..

[bib46] Herrmann I., Pimstein A., Karnieli A., Cohen Y., Alchanatis V., Bonfil D.J. (2011). LAI assessment of wheat and potato crops by VENμS and Sentinel-2 bands. Remote Sens. Environ..

[bib47] Houborg R., Anderson M., Daughtry C. (2009). Utility of an image-based canopy reflectance modeling tool for remote estimation of LAI and leaf chlorophyll content at the field scale. Remote Sens. Environ..

[bib48] Houborg R., McCabe M., Cescatti A., Gao F., Schull M., Gitelson A. (2015). Joint leaf chlorophyll content and leaf area index retrieval from Landsat data using a regularized model inversion system (REGFLEC). Remote Sens. Environ..

[bib49] Huang J., Gómez-Dans J.L., Huang H., Ma H., Wu Q., Lewis P.E., Liang S., Chen Z., Xue J.H., Wu Y., Zhao F., Wang J., Xie X. (2019). Assimilation of remote sensing into crop growth models: current status and perspectives. Agric. For. Meteor..

[bib50] Huang J., Ma H., Sedano F., Lewis P., Liang S., Wu Q., Su W., Zhang X., Zhu D. (2019). Evaluation of regional estimates of winter wheat yield by assimilating three remotely sensed reflectance datasets into the coupled WOFOST–PROSAIL model. Eur. J. Agron..

[bib51] Jacquemoud S., Bacour C., Poilvé H., Frangi J.P. (2000). Comparison of four radiative transfer models to simulate plant canopies reflectance: direct and inverse mode. Remote Sens. Environ..

[bib52] Jacquemoud S., Baret F. (1990). PROSPECT: a model of leaf optical properties spectra. Remote Sens. Environ..

[bib53] Jacquemoud S., Verhoef W., Baret F., Bacour C., Zarco-Tejada P.J., Asner G.P., François C., Ustin S.L. (2009). PROSPECT + SAIL models: A review of use for vegetation characterization. Remote Sens. Environ..

[bib54] Jay S., Maupas F., Bendoula R., Gorretta N. (2017). Retrieving LAI, chlorophyll and nitrogen contents in sugar beet crops from multi-angular optical remote sensing: Comparison of vegetation indices and PROSAIL inversion for field phenotyping. Field Crop. Res..

[bib55] Ji L., Peters A.J. (2007). Performance evaluation of spectral vegetation indices using a statistical sensitivity function. Remote Sens. Environ..

[bib56] Jiang J., Comar A., Burger P., Bancal P., Weiss M., Baret F. (2018). Estimation of leaf traits from reflectance measurements: Comparison between methods based on vegetation indices and several versions of the PROSPECT model. Plant Methods.

[bib57] Jonckheere I., Fleck S., Nackaerts K., Muys B., Coppin P., Weiss M., Baret F. (2004). Review of methods for in situ leaf area index determination Part I. Theories, sensors and hemispherical photography. Agric. For. Meteorol..

[bib58] Kayad A., Sozzi M., Gatto S., Marinello F., Pirotti F. (2019). Monitoring within-field variability of corn yield using sentinel-2 and machine learning techniques. Remote Sens..

[bib59] Kayad A., Sozzi M., Gatto S., Whelan B., Sartori L., Marinello F. (2021). Ten years of corn yield dynamics at field scale under digital agriculture solutions: a case study from North Italy. Comput. Electron. Agric..

[bib60] Kayad A.G., Al-Gaadi K.A., Tola E., Madugundu R., Zeyada A.M., Kalaitzidis C. (2016). Assessing the spatial variability of alfalfa yield using satellite imagery and ground-based data. PLoS One.

[bib61] Kimm H., Guan K., Jiang C., Peng B., Gentry L.F., Wilkin S.C., Wang S., Cai Y., Bernacchi C.J., Peng J., Luo Y. (2020). Deriving high-spatiotemporal-resolution leaf area index for agroecosystems in the U.S. Corn Belt using Planet Labs CubeSat and STAIR fusion data. Remote Sens. Environ..

[bib62] Knipling E. (1970). Physical and physiological basis for the reflectance of visible and near-infrared radiation from vegetation. Remote Sens. Environ..

[bib63] Lambert M.J., Traoré P.C.S., Blaes X., Baret P., Defourny P. (2018). Estimating smallholder crops production at village level from Sentinel-2 time series in Mali’s cotton belt. Remote Sens. Environ..

[bib64] Lan Y., Thomson S.J., Huang Y., Hoffmann W.C., Zhang H. (2010). Current status and future directions of precision aerial application for site-specific crop management in the USA. Comput. Electron. Agric..

[bib65] Lobell D.B. (2013). The use of satellite data for crop yield gap analysis. F. Crop. Res..

[bib66] Madugundu R., Al-Gaadi K., Tola E., Kayad A., Jha C. (2017). Estimation of gross primary production of irrigated maize using Landsat-8 imagery and Eddy Covariance data. Saudi J. Biol. Sci..

[bib67] Mananze S., Pôças I., Cunha M. (2018). Retrieval of maize leaf area index using hyperspectral and multispectral data. Remote Sens..

[bib68] Matsushita B., Yang W., Chen J., Onda Y., Qiu G. (2007). Sensitivity of the Enhanced Vegetation Index (EVI) and Normalized Difference Vegetation Index (NDVI) to topographic effects: a case study in high-density cypress forest. Sensors.

[bib69] Meisner, C.A., Acevedo, E., Flores, D., Sayre, K.D., Ortiz-Monasterio, I., Byerlee, D., 1992. Wheat production and grower practices in the Yaqui Valley, Sonora, Mexico. Wheat Special Report No. 6.

[bib70] Mkhabela M.S., Bullock P., Raj S., Wang S., Yang Y. (2011). Crop yield forecasting on the Canadian Prairies using MODIS NDVI data. Agric. Meteorol..

[bib71] Monteith J.L. (1972). Solar radiation and productivity in tropical ecosystems. J. Appl. Ecol..

[bib72] Nguy-Robertson A., Gitelson A., Peng Y., Viña A., Arkebauer T., Rundquist D. (2012). Green leaf area index estimation in maize and soybean: Combining vegetation indices to achieve maximal sensitivity. Agron. J..

[bib73] Peralta N.R., Assefa Y., Du J., Barden C.J., Ciampitti I.A. (2016). Mid-season high-resolution satellite imagery for forecasting site-specific corn yield. Remote Sens..

[bib74] Punalekar S.M., Verhoef A., Quaife T.L., Humphries D., Bermingham L., Reynolds C.K. (2018). Application of Sentinel-2A data for pasture biomass monitoring using a physically based radiative transfer model. Remote Sens. Environ..

[bib75] Qiao K., Zhu W., Xie Z. (2020). Application conditions and impact factors for various vegetation indices in constructing the LAI seasonal trajectory over different vegetation types. Ecol. Indic..

[bib76] Rembold F., Atzberger C., Savin I., Rojas O. (2013). Using low resolution satellite imagery for yield prediction and yield anomaly detection. Remote Sens.

[bib77] Richter K., Atzberger C., Vuolo F., D’Urso G. (2011). Evaluation of sentinel-2 spectral sampling for radiative transfer model based LAI estimation of wheat, sugar beet, and maize. IEEE J. Sel. Top. Appl. Earth Obs. Remote Sens..

[bib78] Richter K., Atzberger C., Vuolo F., Weihs P., D’urso G. (2009). Experimental assessment of the Sentinel-2 band setting for RTM-based LAI retrieval of sugar beet and maize. Can. J. Remote Sens..

[bib79] Rivera J.P., Verrelst J., Delegido J., Veroustraete F., Moreno J. (2014). On the semi-automatic retrieval of biophysical parameters based on spectral index optimization. Remote Sens.

[bib80] Rodrigues F.A., Blasch G., Defourny P., Ortiz-Monasterio J.I., Schulthess U., Zarco-Tejada P.J., Taylor J.A., Gérard B. (2018). Multi-temporal and spectral analysis of high-resolution hyperspectral airborne imagery for precision agriculture: assessment of wheat grain yield and grain protein content. Remote Sens..

[bib81] Schulthess U., Timsina J., Herrera J.M., McDonald A. (2013). Mapping field-scale yield gaps for maize: an example from Bangladesh. Field Crop. Res..

[bib82] Schwalbert R.A., Amado T.J.C., Nieto L., Varela S., Corassa G.M., Horbe T.A.N., Rice C.W., Peralta N.R., Ciampitti I.A. (2018). Forecasting maize yield at field scale based on high-resolution satellite imagery. Biosyst. Eng..

[bib83] Scotford I.M., Miller P.C.H. (2005). Applications of spectral reflectance techniques in northern European cereal production: a review. Biosyst. Eng..

[bib84] Sehgal V.K., Chakraborty D., Sahoo R.N. (2016). Inversion of radiative transfer model for retrieval of wheat biophysical parameters from broadband reflectance measurements. Inf. Process. Agric..

[bib85] Shanahan J.F., Schepers J.S., Francis D.D., Varvel G.E., Wilhelm W.W., Tringe J.M., Schlemmer M.R., Major D.J. (2001). Use of remote-sensing imagery to estimate corn grain yield. Agron. J..

[bib86] Si Y., Schlerf M., Zurita-Milla R., Skidmore A., Wang T. (2012). Mapping spatio-temporal variation of grassland quantity and quality using MERIS data and the PROSAIL model. Remote Sens. Environ..

[bib87] Strachan I.B., Stewart D.W., Pattey E. (2015). Micrometeorology in Agricultural Systems.

[bib88] Su W., Huang J., Liu D., Zhang M. (2019). Retrieving corn canopy leaf area index from multitemporal landsat imagery and terrestrial LiDAR data. Remote Sens..

[bib89] Su W., Sun Z., Chen W.H., Zhang X., Yao C., Wu J., Huang J., Zhu D. (2019). Joint retrieval of growing season corn canopy LAI and leaf chlorophyll content by fusing Sentinel-2 and MODIS images. Remote Sens..

[bib90] Towers P.C., Strever A., Poblete-Echeverría C. (2019). Comparison of vegetation indices for leaf area index estimation in vertical shoot positioned vine canopies with and without grenbiule hail-protection netting. Remote Sens..

[bib91] Trotter, T., Frazier, P., Trotter, M., Lamb, D., 2008. Objective biomass assessment using an active plant sensor (crop circleTM)- preliminary experiences on a variety of agricultural landscapes, in: 9th International Conference on Precision Agriculture (ICPA). Denver, Colorado, USA.

[bib92] Venancio L.P., Mantovani E.C., Amaral C.H., do, Neale C.M.U., Gonçalves I.Z., Filgueiras R., Eugenio F.C. (2020). Potential of using spectral vegetation indices for corn green biomass estimation based on their relationship with the photosynthetic vegetation sub-pixel fraction. Agric. Water Manag..

[bib93] Verhoef W. (1985). Earth observation modeling based on layer scattering matrices. Remote Sens. Environ..

[bib94] Verhoef W. (1984). Light scattering by leaf layers with application to canopy reflectance modeling: The SAIL model. Remote Sens. Environ..

[bib95] Verrelst J., Rivera J.P., Gitelson A., Delegido J., Moreno J., Camps-Valls G. (2016). Spectral band selection for vegetation properties retrieval using Gaussian processes regression. Int. J. Appl. Earth Obs. Geoinf..

[bib96] Verrelst J., Romijn E., Kooistra L. (2012). Mapping vegetation density in a heterogeneous river floodplain ecosystem using pointable CHRIS/PROBA data. Remote Sens..

[bib97] Weiss M., Baret F., Smith G.J., Jonckheere I., Coppin P. (2004). Review of methods for in situ leaf area index (LAI) determination Part II. Estimation of LAI, errors and sampling. Agric. For. Meteorol..

[bib98] Yao Y., Liu Qinhuo, Liu, Qiang Li, X. (2008). LAI retrieval and uncertainty evaluations for typical row-planted crops at different growth stages. Remote Sens. Environ..

[bib99] Zarco-Tejada P.J., González-Dugo V., Berni J.A.J. (2012). Fluorescence, temperature and narrow-band indices acquired from a UAV platform for water stress detection using a micro-hyperspectral imager and a thermal camera. Remote Sens. Environ..

[bib100] Zarco-Tejada P.J., González-Dugo M.V., Fereres E. (2016). Seasonal stability of chlorophyll fluorescence quantified from airborne hyperspectral imagery as an indicator of net photosynthesis in the context of precision agriculture. Remote Sens. Environ..

[bib101] Zhang L., Guo C.L., Zhao L.Y., Zhu Y., Cao W.X., Tian Y.C., Cheng T., Wang X. (2016). Estimating wheat yield by integrating the WheatGrow and PROSAIL models. Field Crop. Res..

